# Competition Between Conjugation and M13 Phage Infection in *Escherichia coli* in the Absence of Selection Pressure: A Kinetic Study

**DOI:** 10.1534/g3.112.003418

**Published:** 2012-10-01

**Authors:** Zhenmao Wan, Noel L. Goddard

**Affiliations:** * Department of Physics & Astronomy, Hunter College, City University of New York, New York 10065, and; † Department of Physics, Graduate Center, City University of New York, New York 10016; ‡ Department of Biology, Graduate Center, City University of New York, New York 10016

**Keywords:** conjugation, qPCR, M13 phage, phage kinetics, evolution of sex

## Abstract

Inter- and intraspecies horizontal gene transfer enabled by bacterial secretion systems is a powerful mechanism for bacterial genome plasticity. The type IV secretion system of *Escherichia coli*, encoded by the F plasmid, enables cell-to-cell contact and subsequent DNA transfer known as conjugation. Conjugation is compromised by phage infection that specifically targets the secretion machinery. Hence, the use of phages to regulate the spread of genes, such as acquired antibiotic resistance or as general biosanitation agents, has gained interest. To predict the potential efficacy, the competition kinetics must first be understood. Using quantitative PCR to enumerate genomic loci in a resource-limited batch culture, we quantify the infection kinetics of the nonlytic phage M13 and its impact on conjugation in the absence of selection pressure (isogenic set). Modeling the resulting experimental data reveals the cellular growth rate to be reduced to 60% upon phage infection. We also find a maximum phage infection rate of 3×10^−11^ mL phage^−1^ min^−1^ which is only 1 order of magnitude slower than the maximum conjugation rate (3×10^−10^ mL cell^−1^ min^−1^), suggesting phages must be in significant abundance to be effective antagonists to horizontal gene transfer. In the regime where the number of susceptible cells (F^+^) and phages are equal upon initial infection, we observe the spread of the conjugative plasmid throughout the cell population despite phage infection, but only at 10% of the uninfected rate. This has interesting evolutionary implications, as even in the absence of selection pressure, cells maintain the ability to conjugate despite phage vulnerability and the associated growth consequences.

Bacteriophages are the most abundant organism on earth (Bergh *et al.* 1989), estimated to outnumber bacteria by an order of magnitude. They were first described in the early part of the twentieth century by two independent scientists (d’Herelle 1917; Twort 1915) who found they were agonists to bacteria, resulting in lysis and cell death. Phages later became instrumental to the burgeoning field of molecular genetics in which they contributed to the understanding that DNA was the genetic material (Hershey and Chase 1952), they were used to elucidate the rate of spontaneous mutation (Luria and Delbruck 1943), they facilitated the discovery of mRNA (Volkin and Astrachan 1956), and they enabled the isolation of the first DNA binding proteins (Gilbert and Muller-Hill 1966; Ptashne 1967). In the genomics era, small phage genomes were the first to be sequenced (Fiers *et al.* 1976; Sanger *et al.* 1977), while prophages would later lay the foundation for whole-genome sequencing of bacteria (Blattner *et al.* 1997; Burland *et al.* 1993). Due to their nanoscale, ease of genetic manipulation, and self-assembly characteristics, phages are increasingly used for biotechnological applications (Petty *et al.* 2007) of peptide library expression, protein panning, pathogen detection, and therapeutic delivery devices (Clark and March 2006).

Conjugation was first observed in the gram-negative species *Escherichia coli* (Lederberg and Tatum 1946) and later in the gram-positive species *Streptococcus faecalis* (Dunny *et al.* 1978). Conjugative plasmids (Novick *et al.* 1976) encode for the necessary components for mating pair formation and subsequent DNA transfer. They are a powerful tool for genome evolution as they can harbor and transfer genes between organisms, sampling all genomes within an ecosystem (Norman *et al.* 2009). Genomic evidence reveals DNA transfer between genera, phyla, and even major domains (Slater *et al.* 2008; van Elsas and Bailey 2002).

Cells containing the conjugative plasmid are referred to as donors, and the cells receiving the plasmid are termed recipients. Following mating pair formation, the conjugative plasmid is transferred to the recipient, thus enabling the recipient to become an active donor. The mating bridge allows the two genomes to participate in sexual recombination (Lederberg and Tatum 1946), as well as transferring any smaller nonconjugative plasmids (Andrup *et al.* 1996). Like other plasmids, conjugative plasmids contain a replication origin, recruiting the host polymerases to replicate and thus propagate the plasmid to daughter cells through replication as well. This muddles the boundary between proliferation and genetic exchange in prokaryotes (Levin and Bergstrom 2000).

In this work, we focus on a model conjugative system, the F plasmid in *E. coli* (Lederberg *et al.* 1952), which encodes for a Type IV secretion system. Conjugation commences when the tips of F pili from a donor cell (termed F^+^) make contact with recipient cells (F^−^) (Novik *et al.* 1976; Norman *et al.* 2009), creating a mating bridge. Recent work has shown the pili to be dynamic structures, extending and retracting continuously (Clarke *et al.* 2008). When the pili retract, the cells form a mating pair aggregate (Achtman 1975). The filamentous phage M13 targets the tip of the pili, inhibiting conjugation (Novotny *et al.* 1968; Ou 1973). Following infection with M13, the *E. coli* host continues to grow and divide, continuously secreting phage particles (Aksyuk and Rossmann 2011; Russel 1991).

Concerns about horizontal gene transfer in the clinical environment, enabling acquired antibiotic resistance (Chavers *et al.* 2003; Grohmann *et al.* 2003), have motivated studies on the use of phages or their enzymes (Fischetti *et al.* 2006) for biosanitation. Likewise, the widespread use of antibiotics in agriculture has created antibiotic-resistant ‘super-bugs’, therefore demanding alternative methods such as the use of phages for pathogen control (Mahony *et al.* 2011). As these technologies mature, it is crucial to better understand the kinetics of phage infection to predict their propagation, persistence, and efficacy. Recent studies (Lin *et al.* 2011) have addressed the use of phages to inhibit horizontal transfer and the dynamics of clonal variability in viral spread (De Paepe *et al.* 2010). We present a complementary study in which the population of phages and susceptible cells mimics the estimated environmental regime to understand the maintenance of conjugation in natural bacterial populations.

## Materials and Methods

### Strains

W6 (F^+^, *relA1 spoT1 metB1 rrnB-2 creC5-10*), an early derivative of the original K12 (F^+^) strain (Lederberg *et al.* 1952), was used as both donor and recipient. The strains were acquired from the Yale Coli Genetic Stock Center. We created a W6 (F^−^) by “curing” W6 (F^+^) with a modified (Wan *et al.* 2011) acridine orange protocol (Hirota 1960). After curing, we sequenced *rpoH*, a gene essential to F plasmid replication (Wada *et al.* 1987), to ensure the absence of secondary mutations. Although the choice of a *relA spoT* background may influence the growth of the cells in media shift experiments, there is evidence that *relA spoT* mutants have comparable growth rates to *relA+ spoT+* and that the mutations do not influence F plasmid maintenance (Winkler *et al.* 1979). The choice of W6 was motivated by the desire to study the maintenance of a natural F factor *vs.* engineered F′ episomes under selection.

The M13 bacteriophages (ATCC 15669-B1) were revived by mixing with log phase W6 (F^+^) cells in Luria broth, and they were allowed to grow overnight. Cells were pelleted, and the remaining supernatant was used for viable phage stock. The phages were revived prior to each growth experiment and plated on top of agar at the time of the experiment to measure the titer.

### Growth assays

The growth assays differed based on the three tested conditions: 1) conjugation without phages, 2) no conjugation with phages, and 3) conjugation with phages.

Single colonies of W6 (F^+^) and W6 (F^−^) cells were inoculated into separate Luria broth (∼5 mL) overnight cultures. M13 phages were revived by mixing with log phase W6 (F^+^) cells in Luria broth and allowed to grow overnight. One milliliter of the overnight culture was centrifuged at 12,000 rpm for 10 min (Eppendorf Centrifuge 5417R; Eppendorf AG, Hamburg, Germany). The fresh supernatant containing the revived M13 phages was collected for use in the growth experiments. Prior to each growth experiment, the phages were titered on *E. coli* W6 (F^+^) lawns to quantify the viable concentration used in each experiment.

#### Condition 1) Conjugation without phages:

The overnight W6 (F^+^) and W6 (F^−^) cultures were then mixed in ratios of F^+^:F^−^ [1:1 (500 μL:500 μL), 1:10 (100 μL:1000 μL), 1:10^2^ (10 μL:1000 μL), 1:10^3^ (1 μL:1000 μL)] at room temperature (∼23°). To maintain the same inoculation density in all experiments, 200 μL of each mixture was then used to inoculate 50 mL of preheated (37°) Luria broth in 250 mL flasks (final concentration ∼10^6^ cells mL^−1^).

#### Condition 2A) Constant phage inoculum concentration with variable cell inoculum concentration:

Five hundred microliters of separate 10-fold serial dilutions (10^0^, 10^−1^, and 10^−2^) were prepared from a saturated overnight culture of W6 (F^+^) cells at room temperature (∼23°). Each serial dilution was then mixed with 5 μL of freshly revived M13 phages (titer ∼10^11^ phages mL^−1^). Care was taken to inoculate phage into all dilutions simultaneously, serving as time point 0. The full volume of the cell/phage mixture (505 μL) was then used to inoculate 50 mL of preheated (37°) Luria broth in 250 mL flasks.

#### Condition 2B) Constant cell inoculum concentration with variable phage inoculum concentration:

Ten-fold serial dilutions were prepared from the revived phage supernatant (10^0^, 10^−1^, 10^−2^, 10^−3^, 10^−4^) at room temperature (∼23°). Fifty microliters of each phage dilution were mixed with 50 μL of W6 (F^+^) cells, and the full volume of the mixture (100 μL) was used to inoculate 50 mL of preheated (37°) Luria broth in 250 mL flasks.

#### Condition 2C) Growth of pre-infected F^+^ cells:

An overnight culture of W6 (F^+^) cells were inoculated with M13 phages. Serial dilutions (10^0^, 10^−1^, and 10^−2^) were prepared from the infected overnight culture at room temperature (∼23°). Two hundred microliters of each serial dilution was then used to inoculate 50 mL of preheated (37°) Luria broth in 250 mL flasks.

#### Condition 3) Competition of conjugation and phage infection:

We followed the above procedures in condition 1 for preparation of the F^+^/F^−^ mixtures and prepared serial dilutions of the F+/F^−^ mixtures. Two hundred microliters of each serial dilution was then used to inoculate 50 mL of preheated (37°) Luria broth in 250 mL flasks. At time point 0, each flask was inoculated with 5 μL of a freshly revived phage culture (titer ∼10^11^ phages mL^−1^).

All experiments used 250 mL flasks placed in a water bath immersion shaker (model G67; New Brunswick Scientific, New Brunswick, NJ), shaken at 110 rpm, and maintained at 37°. The rate of conjugal mating pair formation has been shown to be constant over a broad range of shake flask shear forces (0–300 rpm) (Zhong *et al.* 2010). One hundred microliter aliquots were extracted every 15 min or 20 min without pausing the shaking or removing the flasks from the immersion bath. The 100 μL aliquot was diluted into 400 μL water (MilliQ RO purified; Millipore), and then placed in a 95° dry bath to lyse the cells (preserving the cell and phage number and denaturing potential degradation enzymes). Aliquots for the time series were then stored at −20° until their use as templates for the qPCR assays.

### Quantitative PCR assay

All qPCR assays used a master mix consisting of final concentration: 2 mM MgCl_2_, 200 µM dNTP mix, 1U (per 25 μL volume) Roche FastStart Enzyme blend (Roche Diagnostics, Mannheim, Germany), 1× Roche FastStartBuffer (Roche Diagnostics), 0.4 μM forward and reverse primers, 2 μM SYTO 9 (Life Technologies, Carlsbad, CA), and 1X ROX reference dye (Life Technologies). We use SYTO9 dye for double stranded DNA quantification, as it has been shown to have fewer sequence and concentration artifacts (Gudnason *et al.* 2007). Five microliters of the lysed frozen aliquots was used as template for the qPCR reactions (per 25 μL reaction).

All reactions were performed in a BioRad Chromo4 Instrument (Bio-Rad Laboratories, Hercules, CA), in 96-well, clear-bottom, hard-shell, skirted assay plates (Bio-Rad Laboratories) with Microseal B sealing tape (Bio-Rad Laboratories). The instrument filter settings were set for FAM and ROX for SYTO9 and ROX, respectively, where ROX was used as a passive reference.

The Ct values were extracted from the qPCR data as described in our previous work (Wan *et al.* 2011) to quantify the abundance of genomic loci throughout the growth of each culture.

Primers were designed to allow us to measure the growth kinetics of three genetic loci: the *E. coli* chromosome (tolC), the F plasmid (traI), and the M13 phage (M13) (see Table 1). tolC data represent the total number of F^+^ and F^−^ cells, traI data represents the number of F^+^ cells only, and M13 data represents the number of M13 phages.

**Table 1:** Primers and thermal program

Primers	5′→3′	Thermal Program	
tolC forward	CGACAAACCACAGCCGGTTA	95° for 6 min	1 cycle
tolC reverse	CAGCGAGAAGCTCAGGCCA	95° for 30 s	Repeat for 35 cycles
traI forward	GCCATTCATCTTGCCCTTCC	50° for 30 s	
traI reverse	GCATGACCGCCTCCTTACC	Plate read	
M13 forward	TTGTTCCTTTCTATTCTCACTCC	72° for 25 s	
M13 reverse	CACCCTCAGAACCGCCACC		

### Mathematical model

**Figure 1:**
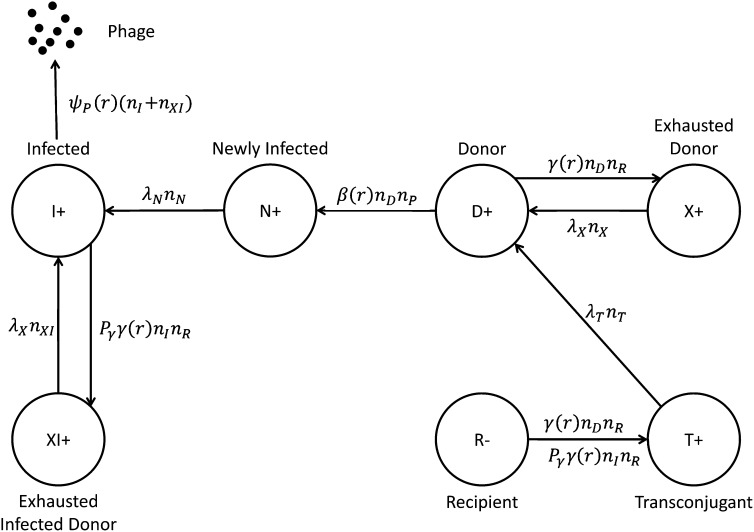
Schematic of the allowed transitions between cellular states for conjugation and phage infection for the mathematical model.

In a previous work (Wan *et al.* 2011), we extracted the plasmid transfer rate during conjugation for a neutral selection condition (isogenic F^+^/F^−^ pair) using a resource-limited logistic growth (hyperbolic) model, similar to the approach of Stewart and Levin (1977). We slightly altered the model from the recent publication, explicitly including the carrying capacity in the resource-limit equation (Equation 1 below). Figure 1 is a schematic describing the all states and allowed transitions between the states used in the model.

During conjugation, recipient cells (R) receive a copy of plasmid from donor cells (D) and become transconjugant cells (T), whereas donor cells become temporarily exhausted donor cells (X). The transit times for transconjugant cells and temporarily exhausted donor cells to become active donor cells are 1/*λ_T_* and 1/*λ_X_*, respectively. Literature values (Andrup and Andersen 1999) for *λ_T_* and *λ_X_* were used in the final fit.

The interaction kinetics becomes more complex with the addition of phage. As M13 phage (P) is a F^+^-specific phage, it can only infect plasmid-bearing donor cells (D) by binding to the tips the pili of F^+^ cells. Newly infected cells (N) cannot produce phages immediately; hence, we introduce a lag time of *λ_N_* for them to become active phage-producing infected cells (I).

When M13 phages (P) are introduced into a mixture of plasmid-bearing (D) and plasmid-free (R) cells, there is competition between conjugation and phage infection. Infected cells (N) can conjugate and become temporarily exhausted infected cells (XI), assuming they have the same lag time of 1/*λ_X_* as uninfected cells.

A schematic of the various states of phages and cells, including all transition pathways, are shown in Figure 1. The dynamics of resource consumption and each cellular state follows (Equations 1–9):

(1) $$\dot{r}=-e\psi \left(r\right)\left(n_{D}+n_{R}+n_{T}+n_{X}\right)\left(1-\frac{\Sigma n}{K}\right)-eP_{\psi }\psi \left(r\right)\left(n_{N}+n_{I}+n_{XI}\right)\left(1-\frac{\Sigma n}{K}\right)$$

(2) $${\dot{n}}_{D}=\psi \left(r\right)n_{D}\left(1-\frac{\Sigma n}{K}\right)-\gamma \left(r\right)n_{D}n_{R}+\lambda_{X}n_{X}+\lambda_{T}n_{T}-\beta \left(r\right)n_{D}n_{P}$$

(3) $${\dot{n}}_{R}=\psi \left(r\right)n_{R}\left(1-\frac{\Sigma n}{K}\right)-\gamma \left(r\right)n_{D}n_{R}-P_{\gamma }\gamma \left(r\right)n_{I}n_{R}$$

(4) $${\dot{n}}_{T}=\psi \left(r\right)n_{T}\left(1-\frac{\Sigma n}{K}\right)+\gamma \left(r\right)n_{D}n_{R}-\lambda_{T}n_{T}+P_{\gamma }\gamma \left(r\right)n_{I}n_{R}$$

(5) $${\dot{n}}_{X}=\psi \left(r\right)n_{X}\left(1-\frac{\Sigma n}{K}\right)+\gamma \left(r\right)n_{D}n_{R}-\lambda_{X}n_{X}$$

(6) $${\dot{n}}_{N}=P_{\psi }\psi \left(r\right)n_{N}\left(1-\frac{\Sigma n}{K}\right)+\beta \left(r\right)n_{D}n_{P}-\lambda_{N}n_{N}$$

(7) $${\dot{n}}_{I}=P_{\psi }\psi \left(r\right)n_{I}\left(1-\frac{\Sigma n}{K}\right)+\lambda_{N}n_{N}-P_{\gamma }\gamma \left(r\right)n_{I}n_{R}+\lambda_{X}n_{XI}$$

(8) $${\dot{n}}_{XI}=P_{\psi }\psi \left(r\right)n_{XI}\left(1-\frac{\Sigma n}{K}\right)+P_{\gamma }\gamma \left(r\right)n_{I}n_{R}-\lambda_{X}n_{XI}$$

(9) $${\dot{n}}_{P}=\psi_{P}\left(r\right)\left(n_{I}+n_{XI}\right)\left(1-\frac{n_{P}}{K_{P}}\right)$$

Equation 1 describes the kinetics where the number of cells in each cellular state (nodes in Figure 1) consumes resources throughout the batch growth assay until it reaches the carrying capacity, *K* (value was determined by using a hemocytometer).

In resource-limited batch culture growth, cells follow logistic growth characterized by the term $\left(1-\frac{\Sigma n}{K}\right)$, where Σ*n* = *n_D_* + *n_R_* + *n_T_* + *n_X_* + *n_N_* + *n_I_* + *n_XI_* is the total number of cells and *K* is the cell carrying capacity. *ψ*(*r*) is the cell growth rate, *P_ψ_* is the growth rate penalty due to phage infection, and the resource consumption of per cell division is *e*.

Equations 2–5 are ordinary differential equations representing the conjugation process. The growth of each population: the number of donors (*n_D_*), recipients (*n_R_*), transconjugants (*n_T_*), and exhausted donors (*n_X_*) are corrected by the number of cells actively conjugating *γ*(*r*)*n_D_n_R_*, where *γ*(*r*) is the conjugation rate. The growth must also correct for the number of cells trapped in states that cannot conjugate (X and T) and are represented by the terms *λ_X_n_X_* and *λ_T_n_T_*, respectively. Following phage inoculation, the number of donors (*n_D_*) has to be corrected by phage infection represented by *β*(*r*)*n_D_n_P_*. Recipients (*n_R_*) and transconjugants (*n_T_*) have to be corrected by conjugation of infected cells (*n_I_*), which is *P_γ_γ*(*r*)*n_I_n_R_*, where *P_γ_* is conjugation rate penalty due to phage infection.

Equations 6–9 describe the transitions between newly infected cells (*n_N_*), infected cells (*n_I_*), exhausted infected donor cells (*n_XI_*), and phages (*n_P_*). Phage particles are continuously produced by infected cells (*n_I_*) and exhausted infected donor cells (*n_XI_*) at a first-order resource-dependent rate *ψ_P_*(*r*) with its own carrying capacity *K_P_*. Similarly, newly infected cells (*n_N_*) are corrected by infected donor cells *β*(*r*)*n_D_n_P_* and the term *λ_N_n_N_* representing the lag time for newly infected donors to become active phage producers (*n_I_*). It is important to note that *β*(*r*) represents the combined efficacy of infection and propagation as our assay cannot distinguish the difference among surface bound, internalized, and secreted phage genomes. Infected cells (*n_I_*) and exhausted infected donor cells (*n_XI_*) are corrected by conjugation *P_γ_γ*(*r*)*n_I_n_R_* and *λ_X_n_XI_*, describing the time an infected donor needs for recovery following conjugation to a recipient cell.

Monod (1942) proposed that bacterial growth kinetics resemble enzyme kinetics in terms of substrate limitation, an idea that was further refined for modeling conjugative transfer as a Michaelis-Menten kinetic scheme (Andrup and Andersen 1999). We assume the cell growth rate *ψ*(*r*), conjugation rate *γ*(*r*), phage infection rate *β*(*r*), and phage production rate *ψ_P_*(*r*) all follow a hyperbolic form similar to Michaelis-Menten kinetics:

(10) $$\psi \left(r\right)=\frac{r\psi_{MAX}}{Q+r}$$

(11) $$\gamma \left(r\right)=\frac{r\gamma_{MAX}}{Q+r}$$

(12) $$\beta \left(r\right)=\frac{r\beta_{MAX}}{Q+r}$$

(13) $$\psi_{P}\left(r\right)=\frac{r\psi_{P_{MAX}}}{Q+r}$$

where the subscript MAX denotes the maximum rate for each of the variables, and *Q* denotes the resource concentration when the rate is half-maximum. In practice, simulations of the data found *β*(*r*) ≈ *β_MAX_* under our experimental conditions because the entire population of host cells is infected well before (>100 min) the resource limit begins to impose saturation conditions.

## Results

As the complete model has many unknown parameters, we utilized the different experimental conditions to selectively fit individual parameters, later used for the full model of competition between infection and conjugation. For all data presented in the following figures, we omitted the error bars between replicate time series for better visual clarity. The range of error for all data points was generally ≤ 1 cycle. Fits were performed with consideration for the real error.

First, to determine the maximum conjugation rate *γ* and resource usage *e*, we implemented the experiments on a system without phages (condition 1 in *Materials and Methods*). This eliminates all phage contribution to the kinetics. Equations 1–5 can be simplified to Equations 14–18 as follows:

(14) $$\dot{r}=-e\psi \left(r\right)\left(n_{D}+n_{R}+n_{T}+n_{X}\right)\left(1-\frac{\Sigma n}{K}\right)$$

(15) $${\dot{n}}_{D}=\psi \left(r\right)n_{D}\left(1-\frac{\Sigma n}{K}\right)-\gamma \left(r\right)n_{D}n_{R}+\lambda_{X}n_{X}+\lambda_{T}n_{T}$$

(16) $${\dot{n}}_{R}=\psi \left(r\right)n_{R}\left(1-\frac{\Sigma n}{K}\right)-\gamma \left(r\right)n_{D}n_{R}$$

(17) $${\dot{n}}_{T}=\psi \left(r\right)n_{T}\left(1-\frac{\Sigma n}{K}\right)+\gamma \left(r\right)n_{D}n_{R}-\lambda_{T}n_{T}$$

(18) $${\dot{n}}_{X}=\psi \left(r\right)n_{X}\left(1-\frac{\Sigma n}{K}\right)+\gamma \left(r\right)n_{D}n_{R}-\lambda_{X}n_{X}$$

**Figure 2:**
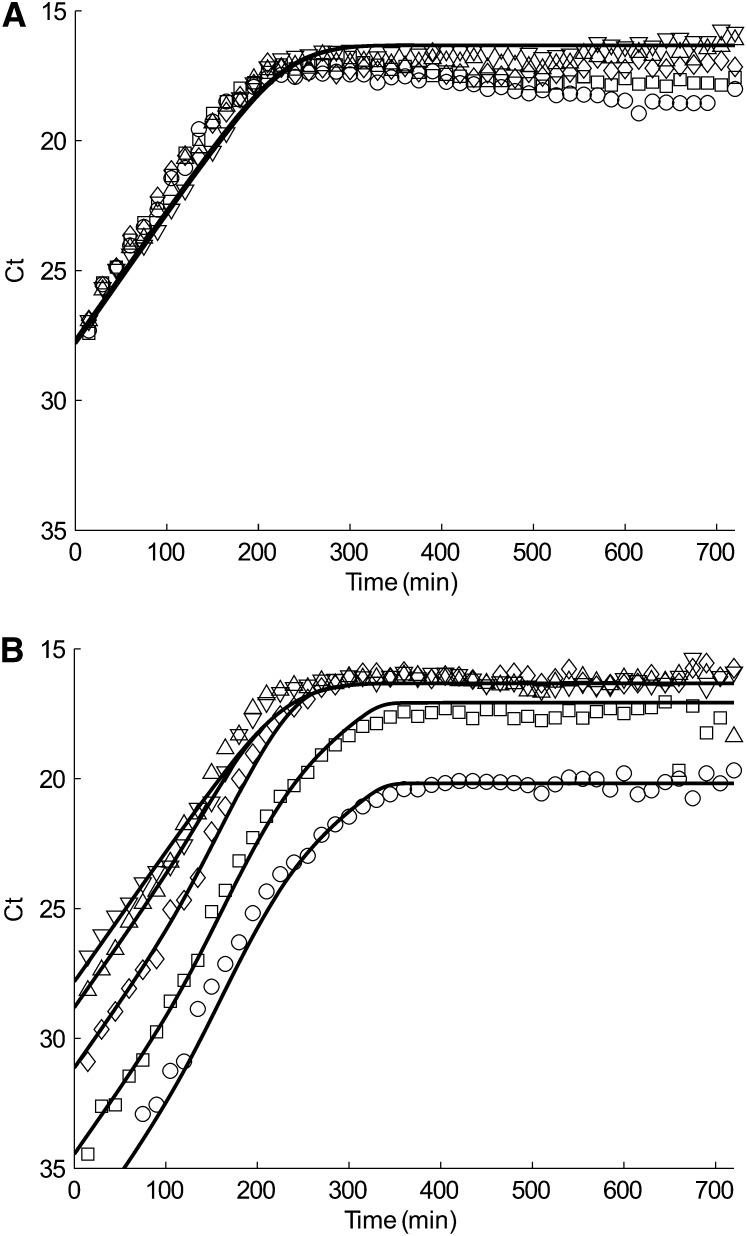
(A) tolC and (B) traI simulation (solid line) and quantitative PCR data (closed symbol) of conjugation-only experiment. Each plot represents a different inoculation ratio of donors to recipients: Pure F+ (donor) culture (downward-pointing triangle), 1:1 (upward-pointing triangle), 1:10 (diamond), 1:10^2^ (square), and 1:10^3^ (circle).

*λ_T_* = 1/90 min^−1^ and *λ_X_* = 1/30 min^−1^ are taken from the literature (Andrup and Andersen 1999; Cullum *et al.* 1978). We estimate the cell-carrying capacity, *K* = 3×10^9^ cells mL^−1^, by counting the number of cells at saturation using a hemocytometer. Because *r* scales with *e* (Equation 14), we chose to define them in terms of arbitrary units. Allowing *Q* = 1 arbitrary unit (a.u.) and *r* = 100 a.u., *e* = 3.5×10^−8^ a.u. mL cell^−1^ and the maximum cell growth rate can be found by fitting the pure F^+^ (no conjugation) growth curve (downward-pointing triangle in Figure 2). The value of *e* for Luria broth is about 2-fold greater than the reported value from our previous work (Wan *et al.* 2011) as we mathematically redefined the expression for carrying capacity. We find *ψ_MAX_* = 0.035 min^−1^, similar to the reported value (Berney *et al.* 2006) (autoclaved Luria broth medium ∼2 h^−1^ = 0.033 min^−1^).

After empirically establishing the parameters *K*, *e*, and *ψ_MAX_* at fixed *λ_T_* and *λ_X_*, we could use the ratio experiments of F^+^/F^−^ conjugation (condition 1 methods) to fit *γ_MAX_*. Figure 2 displays the data and fits for the chromosomal marker tolC (Figure 2A) and F plasmid marker traI (Figure 2B) from different mixture experiments of donor:recipient cells, 1:1 (upward-pointing triangle), 1:10 (diamond), 1:10^2^ (square), and 1:10^3^ (circle). We find *γ_MAX_* = 3×10^−10^ mL cell^−1^ min^−1^, which is slightly lower than our previously reported value (Wan *et al.* 2011) of 5×10^−10^ mL cell^−1^ min^−1^ due to the change in the mathematical expression for the resource limit (Equation 1).

Next, we consider phage infection without conjugation (condition 2 in *Materials and Methods*). The relevant Equations 1, 2, 6, 7 and 9 can be simplified as follows:

(19) $$\dot{r}=-e\psi \left(r\right)n_{D}\left(1-\frac{\Sigma n}{K}\right)-eP_{\psi }\psi \left(r\right)\left(n_{N}+n_{I}+n_{XI}\right)\left(1-\frac{\Sigma n}{K}\right)$$

(20) $${\dot{n}}_{D}=\psi \left(r\right)n_{D}\left(1-\frac{\Sigma n}{K}\right)-\beta \left(r\right)n_{D}n_{P}$$

(21) $${\dot{n}}_{N}=P_{\psi }\psi \left(r\right)n_{N}\left(1-\frac{\Sigma n}{K}\right)+\beta \left(r\right)n_{D}n_{P}-\lambda_{N}n_{N}$$

(22) $${\dot{n}}_{I}=P_{\psi }\psi \left(r\right)n_{I}\left(1-\frac{\Sigma n}{K}\right)+\lambda_{N}n_{N}$$

(23) $${\dot{n}}_{p}=\psi_{P}\left(r\right)n_{I}\left(1-\frac{n_{P}}{K_{P}}\right)$$

**Figure 3:**
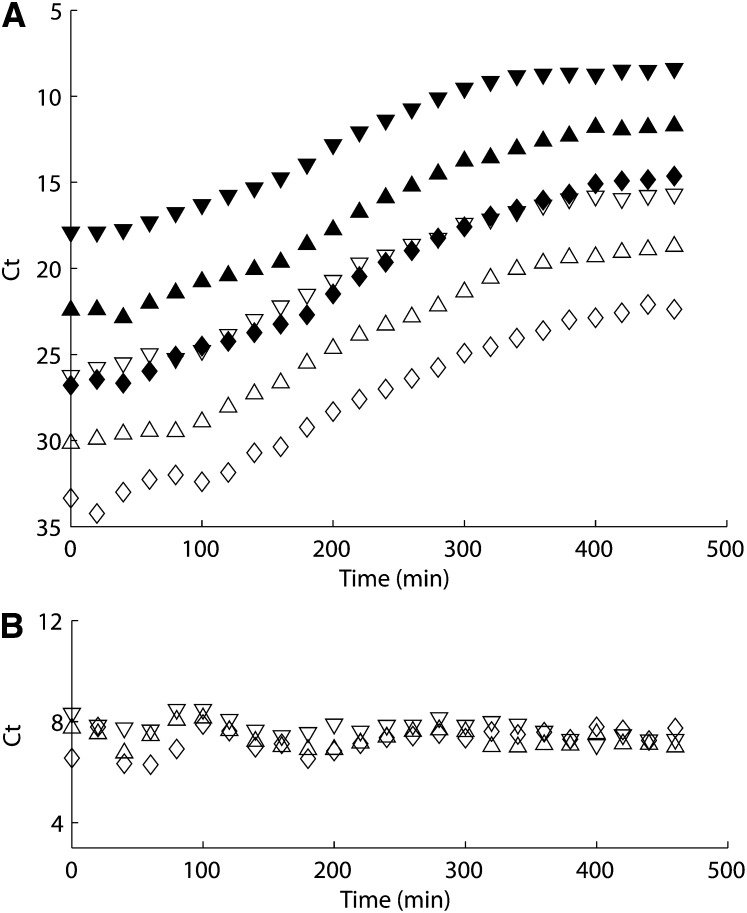
(A) tolC (closed symbol) and M13 (filled symbol) quantitative PCR data of phage pre-infected cells growth experiment of different inoculation concentrations: 10^0^ (downward-pointing triangle), 10^−1^ (upward-pointing triangle), and 10^−2^ (diamond). (B) Difference between tolC and M13 Ct values.

We used a standard plaque assay to estimate the phage-carrying capacity, *K_P_* = 4×10^11^ phages mL^−1^ which is two orders of magnitude greater than the cell-carrying capacity *K* = 3×10^9^ cells mL^−1^. This suggests that each cell can only sustain about 100∼200 M13 phages. To further validate this number, we performed a growth study on pure F^+^ cells that had been pre-infected with M13 the previous day (variant condition 2 in *Materials and Methods*). As we can see from Figure 3A, pre-infected cells and phages grow at similar rates, meaning phage particles are continuously released from cells throughout the growth cycle. In Figure 3B, we plotted time series of the difference between the Ct values of tolC and M13, and we found the Ct value difference is almost constant (7∼8) throughout the whole growth period. This confirms our previous result that each cell can sustain about 2^7^∼2^8^ (128∼256) phages.

**Figure 4:**
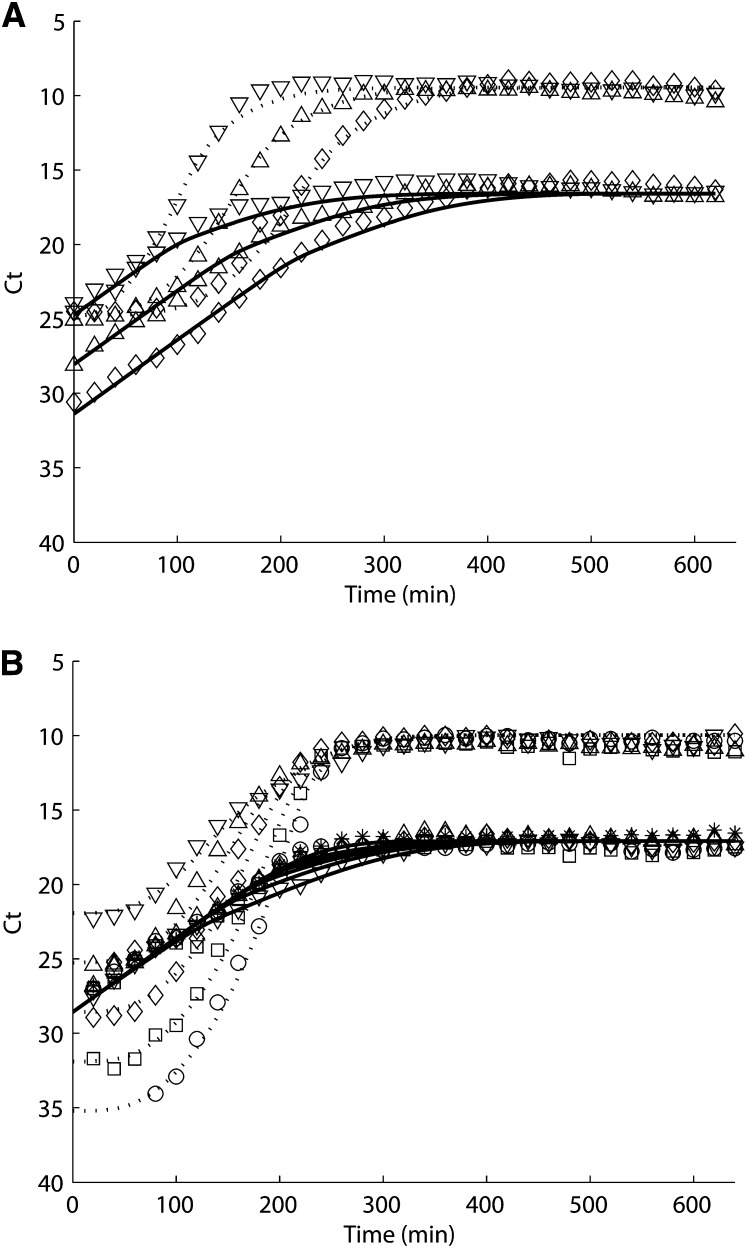
(A) Varying cell concentration and (B) varying phage concentration experiments simulation of tolC (solid line) and M13 (dotted line) and quantitative PCR data (closed symbol). Each plot represents a different inoculation ratio of F+ cells to M13 phages: (A) 1:1 (downward-pointing triangle), 1:10 (upward-pointing triangle), 1:10^2^ (diamond); (B) 1: 10^2^ (downward-pointing triangle), 1:10 (upward-pointing triangle), 1:1 (diamond), 10:1 (square), and 10^2^:1 (circle).

It has been reported that a latent period exists of 30 min at 37° before the burst of phages (Ellis and Delbruck 1939) and that this is equivalent to the delay in newly infected cells becoming phage-producing infected cells, *λ_N_* = 1/30 min^−1^ in our model. F^+^ cells are infected at a rate of *β*(*r*)*n_D_n_P_*, and phages are produced at a rate of *ψ_P_*(*r*)*n_I_* by infected cells. The fitness of infected cells are also reduced (Lin *et al.* 2011) by a penalty factor *P_ψ_*. The maximum phage infection rate *β_MAX_* = 3×10^−11^ mL phage^−1^ min^−1^, the maximum phage production rate $\psi_{P_{MAX}}$ = 6 phages cell^−1^ min^−1^, and the penalty factor of cell growth rate due to infection *P_ψ_* = 0.6 are found by curve-fitting the two different phage infection experiments: Figure 4A, different concentrations of cells (solid line) infected by the same concentration of phages (*dotted line*), 1:1 (downward-pointing triangle), 1:10 (upward-pointing triangle), 1:10^2^ (diamond); and Figure 4B, the same concentration of cells (solid line) infected by different concentrations of phages (*dotted line*), 1:10^2^ (downward-pointing triangle), 1:10 (upward-pointing triangle), 1:1 (diamond), 10:1 (square), and 10^2^:1 (circle).

The trend of phage growth is sensitive to the change of *β_MAX_*, $\psi_{P_{MAX}}$, and *λ_N_*. If increasing any of the parameters, the slope of initial phage growth curve becomes sharper; if decreasing any parameter, it takes a longer time for phages to reach saturation while preserving the relative shape of the curve (figures not shown).

**Figure 5:**
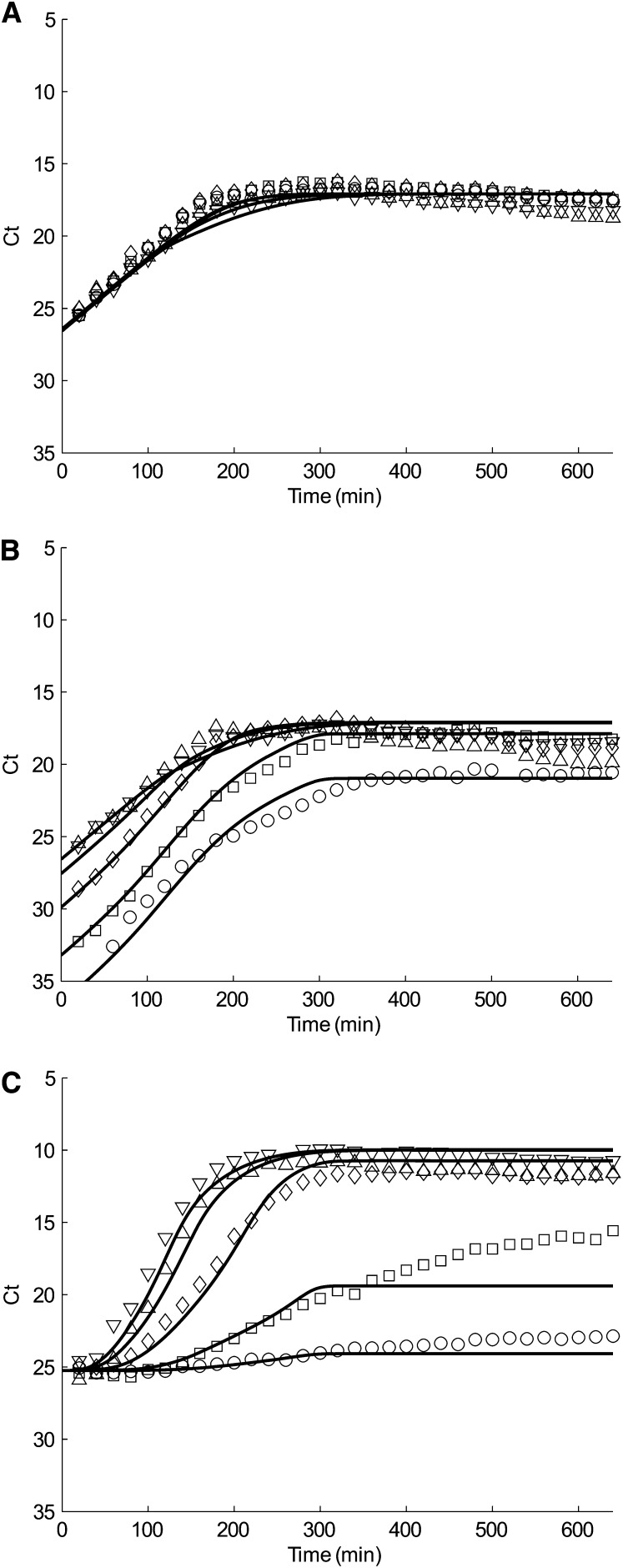
(A) tolC, (B) traI, and (C) M13 simulation (solid line) and quantitative PCR data (closed symbol) of conjugation and phage infection experiment. Each plot represents a different inoculation ratio of donors to recipients: Pure F+ (donor) culture (downward-pointing triangle), 1:1 (upward-pointing triangle), 1:10 (diamond), 1:10^2^ (square), and 1:10^3^ (circle).

Finally, we go back to our original system with mixtures of F^+^ cells, F^−^ cells, and M13 phages. The only unknown parameter is the penalty factor of conjugation rate due to infection (*P_λ_*). The best value we find is *P_λ_* = 0.1 by fitting the data of tolC (Figure 5A), traI (Figure 5B), and M13 (Figure 5C) from our mixture experiments: different ratio of donor:recipient cells, 1:0 (downward-pointing triangle), 1:1 (upward-pointing triangle), 1:10 (diamond), 1:10^2^ (square), and 1:10^3^ (circle) infected by the same concentration of phages. For reference, all parameters used in simulations are summarized in Table 2.

**Table 2:** Parameter values used in simulation

Physical Property	Parameter	Value
Maximum cell growth rate	*ψ_MAX_*	0.035 min^−1^
Maximum conjugation rate	*γ_MAX_*	3×10^−10^ mL cell^−1^ min^−1^
Maximum phage infection rate	*β_MAX_*	3×10^−11^ mL phage^−1^ min^−1^
Maximum phage production rate	$\psi_{P_{MAX}}$	6 phages cell^−1^ min^−1^
Concentration of Resource when rate is half-maximum	*Q*	1 a.u.
Resource consumption per cell division	*e*	3.5×10^−8^ a.u. mL cell^−1^
Cell carrying capacity	*K*	3×10^9^ cells mL^−1^
Phage carrying capacity	*K_P_*	4×10^11^ phages mL^−1^
Delay in transconjugant cells becoming donor cells	*λ_T_*	1/90 min^−1^
Delay in (infected) exhausted donor cells becoming (infected) donor cells	*λ_X_*	1/30 min^−1^
Delay in newly infected cells becoming phage producing infected cells	*λ_N_*	1/30 min^−1^
Penalty factor of cell growth rate due to infection	*P_ψ_*	0.6
Penalty factor of conjugation rate due to infection	*P_λ_*	0.1

a.u., arbitrary unit.

Unlike traI, M13 growth curve (Figure 5C) is very sensitive to the change of *P_λ_*. Increasing *P_λ_* results in underestimation of the growth of M13 phages (data not shown). This is because a higher value of *P_λ_* leads to a larger portion of infected F^+^ to engage in conjugation rather than phage production. Our *P_λ_* = 0.1 fits 1:0, 1:1, and 1:10 mixtures well, but only fits the first 300 min for 1:10^2^ and 1:10^3^ mixtures.

## Discussion

As previously reported (Wan *et al.* 2011), the maximum conjugation rate is of the same order of magnitude as the estimated encounter rate. Hence, conjugation is occurs at maximum efficiency in uninfected cells. Despite the reduced growth rate (60%) induced by phage infection, we find the cells continue to conjugate. Although the efficiency of conjugation is reduced to 10% of its maximum rate, we find the conjugative plasmid still spreads throughout the population to levels that are comparable to uninfected populations (Figure 2B *vs.* Figure 5B). In fact, the simulation of traI is not sensitive to the conjugation penalty factor *P_λ_* upon infection, suggesting that conjugation may occur at the time of mixing (inoculation), before M13 phage attach the pili. To understand the relative encounter frequencies, we can compare the conjugation rate to phage infection rate at the beginning of mixing. In our model, the encounter of donor F^+^ and recipient F^−^ cells results in the conjugation rate of *γ*(*r*)*n_D_n_R_*, whereas the encounter of donor F^+^ cells and M13 phages leads to the infection rate of *β*(*r*)*n_D_n_P_*. The ratio of conjugation rate to phage infection rate is the following:

(24) $$\frac{\gamma \left(r\right)n_{D}n_{R}}{\beta \left(r\right)n_{D}n_{P}}=\frac{\gamma_{MAX}n_{R}}{\beta_{MAX}n_{P}}$$

In the experiments modeled here, *γ_MAX_*/*β_MAX_* = 10, with a population difference of F^−^ to M13 of *n_R_*/*n_P_* ∼ 0.5 upon inoculation. Hence the ratio of conjugation rate to phage infection rate is ∼5, meaning a F^+^ cell has a higher probability of encountering a F^−^ and conjugating than being infected by a M13 phage within the regime defined by our inoculation conditions. Recent kinetic studies (Lin *et al.* 2011) on the use of phages to regulate the conjugative spread of antibiotic resistance markers inoculated in a regime where *n_R_* ≪ *n_P_* by several orders of magnitude found the conditions sufficient for total inhibition of conjugation.

## Conclusion

We demonstrated an experimental assay to measure the growth and competition kinetics between phage infection and conjugation between their bacterial hosts. Simulations of the mathematical model allow us to extract a number of fundamental parameters governing the infection process as well as its inhibitory effect on growth and conjugation. Although conjugation in the environment is frequently under the force of positive selection, we have shown that even in the absence of selective pressure, there is a regime where conjugation can persist despite phage inhibition.
